# PCR-based karyotyping of *Anopheles gambiae *inversion 2*Rj *identifies the BAMAKO chromosomal form

**DOI:** 10.1186/1475-2875-6-133

**Published:** 2007-10-01

**Authors:** Mamadou B Coulibaly, Marco Pombi, Beniamino Caputo, Davis Nwakanma, Musa Jawara, Lassana Konate, Ibrahima Dia, Abdrahamane Fofana, Marcia Kern, Frédéric Simard, David J Conway, Vincenzo Petrarca, Alessandra della Torre, Sékou Traoré, Nora J Besansky

**Affiliations:** 1 Center for Global Health and Infectious Diseases, Department of Biological Sciences, University of Notre Dame, Notre Dame, IN, USA; 2 Malaria Research and Training Center, University of Bamako, Bamako, Mali; 3 Istituto Pasteur-Fondazione Cenci Bolognetti and Dipartimento di Scienze di Sanità Pubblica, Università di Roma "La Sapienza", Rome, Italy; 4 Medical Research Council Laboratories, Fajara, Banjul, The Gambia; 5 Département de Biologie Animale, Faculté des Sciences et Techniques, Université de Dakar, Senegal; 6 Medical Entomology Unit, Pasteur Institute, Dakar, Senegal; 7 Organisation de Coordination pour la Lutte contre les Endémies en Afrique Centrale, Yaoundé, Cameroon; 8 Institut de Recherche pour le Développement, Unité de Recherche 016, Yaoundé, Cameroon; 9 Istituto Pasteur-Fondazione Cenci Bolognetti and Dipartimento di Genetica e Biologia Molecolare, Università di Roma "La Sapienza", Rome, Italy

## Abstract

**Background:**

The malaria vector *Anopheles gambiae *is polymorphic for chromosomal inversions on the right arm of chromosome 2 that segregate nonrandomly between assortatively mating populations in West Africa. One such inversion, 2Rj, is associated with the BAMAKO chromosomal form endemic to southern Mali and northern Guinea Conakry near the Niger River. Although it exploits a unique ecology and both molecular and chromosomal data suggest reduced gene flow between BAMAKO and other *A. gambiae *populations, no molecular markers exist to identify this form.

**Methods:**

To facilitate study of the BAMAKO form, a PCR assay for molecular karyotyping of 2Rj was developed based on sequences at the breakpoint junctions. The assay was extensively validated using more than 700 field specimens whose karyotypes were determined in parallel by cytogenetic and molecular methods. As inversion 2Rj also occurs in SAVANNA populations outside the geographic range of BAMAKO, samples were tested from Senegal, Cameroon and western Guinea Conakry as well as from Mali.

**Results:**

In southern Mali, where 2Rj polymorphism in SAVANNA populations was very low and most of the 2Rj homozygotes were found in BAMAKO karyotypes, the molecular and cytogenetic methods were almost perfectly congruent. Elsewhere agreement between the methods was much poorer, as the molecular assay frequently misclassified 2Rj heterozygotes as 2R+^j ^standard homozygotes.

**Conclusion:**

Molecular karyotyping of 2Rj is robust and accurate on 2R+^j ^standard and 2Rj inverted homozygotes. Therefore, the proposed approach overcomes the lack of a rapid tool for identifying the BAMAKO form across developmental stages and sexes, and opens new perspectives for the study of BAMAKO ecology and behaviour. On the other hand, the method should not be applied for molecular karyotyping of j-carriers within the SAVANNA chromosomal form.

## Background

The mosquito *Anopheles gambiae s.s*. is a widespread and efficient vector of malaria across sub-Saharan Africa. This species also is characterized by abundant chromosomal inversion polymorphisms that are considered important sources of ecological flexibility [1]. Inversion polymorphisms are distributed non-randomly in the *A. gambiae *genome, occurring exclusively on chromosome 2 and predominantly on its right arm (6 of 7 most widely distributed polymorphic inversions, *i.e*. 2Rj, 2Rb, 2Rc, 2Rd, 2Ru, 2Rbk, 2La; [2]). This suggests that the inversions (or genes captured within them) are targets of selection, contributing to more efficient exploitation of the environment and ultimately increased malaria transmission [1].

Not only are inversions distributed non-randomly at the genome level, but also at the population level. In Mali, West Africa, the four most frequent polymorphic inversions (2Rj, b, c, and u) are not found in all possible combinations (karyotypes) even when sampled from the same village, counter to expectation based on random mating. Certain karyotypes are completely absent or significantly reduced in frequency. Based on these data, *A. gambiae *could be subdivided into three assortatively mating chromosomal forms each recognized by characteristic arrangements of 2R: j, b, cu and bcu for SAVANNA; bc and u for MOPTI; jcu and jbcu for BAMAKO [3,4]. Moreover, ecological and physiological differences distinguish chromosomal forms. Whereas SAVANNA is associated with rain-dependent temporary pools far from rivers and irrigated zones and reproductively active only in the rainy season, MOPTI is associated with irrigated agricultural sites and remains reproductively active into the dry season where permanent or semi-permanent larval breeding sites are available. The MOPTI form is distributed mainly in the central part of West Africa, while SAVANNA is broadly distributed in the African savannas, extending east of the Rift Valley. BAMAKO is much more restricted in its range. It is associated with the Niger River and its tributaries in southern Mali and northern Guinea Conakry, and like SAVANNA is reproductively quiescent in the dry season. The individual inversions comprising karyotypic differences between chromosomal forms cannot contain reproductive barriers *per se*, as they are shared between forms by common ancestry as well as ongoing gene flow [4]. However, taken together, the set of 2R arrangements typical of each chromosomal form may reflect the boundaries of different ecological preferences and/or tolerances which can lead to divergence and ultimately speciation.

An important goal driving population genomics studies of *A. gambiae *is to uncover mechanisms for ecological divergence and assortative mating. Addressing those questions requires the development of efficient methods for recognizing inter- and intraspecific subdivisions that do not rely on traditional karyotype analysis – a method limited both by paucity of cytogenetic expertise and availability of properly preserved samples at the correct developmental stage (half-gravid females). In 1997, Favia *et al *[5] reported fixed differences in the X-linked rDNA that distinguish MOPTI from SAVANNA and BAMAKO chromosomal forms in Mali; the latter were indistinguishable at this marker. The fixed rDNA differences defined molecular forms M and S of *A. gambiae*, now considered incipient species [6,7]. The molecular forms are not characterized by form-specific inversion polymorphisms, although these are found at different frequencies in M and S depending on their geographical origin. The only known exception to this general observation concerns the 2Rj inversion, which is exclusive to the S-form according to available records [8]. Within the S molecular form, the 2Rj inversion is present in the homozygous state in association with the 2Rcu arrangement in the chromosomal form BAMAKO (*i.e*., jcu/jcu, jcu/jbcu, jbcu/jbcu), while it may be polymorphic and associated with different chromosomal arrangements in some SAVANNA populations of West Africa [9].

To date, attempts to find fixed molecular differences between SAVANNA and BAMAKO chromosomal forms have failed, though molecular evidence for restricted gene flow exists [10,11]. Based on the fact that the BAMAKO form is characterized by 2Rj in the homozygous state and that sympatric SAVANNA populations harbor only low levels of 2Rj polymorphism, the recently characterized breakpoint junctions of this inversion [12] were exploited to develop a PCR diagnostic assay for molecular determination of the 2Rj karyotype of *A. gambiae *specimens and thus identification of BAMAKO specimens in Mali and other Afrotropical sites where the BAMAKO chromosomal form is prevalent over the 2Rj-carrier SAVANNA. Here, the assay is presented together with the results of extensive validation involving field collections from Mali and three other West and western Central African countries. Samples were karyotyped using traditional and molecular methods in parallel. Overall, molecular and cytogenetic results were almost perfectly congruent for 2Rj standard and inverted homokaryotypes. Thus, in areas of sympatry between BAMAKO and SAVANNA populations with a low frequency of 2Rj (mainly in Mali), molecular karyotyping of 2Rj will be a rapid and effective approach to identify and specifically study the BAMAKO chromosomal form. However, 2Rj inversion heterozygotes were frequently misclassified as 2Rj standard by PCR-based analysis, possibly owing to molecular divergence (null alleles) of 2Rj chromosomes between isolated SAVANNA populations.

## Methods

### Mosquito collections

Collections of indoor resting adults were made by spray catch from seven villages in the southern part of Mali in Aug-Sep 2004: Bancoumana (12°20'N, 8°20'W), Kela (11°88'N, 8°45'W), Banambani (12°48'N, 08°03'W), Moribabougou (12°69'N, 7°87'W), N'Gabakoro (12°68'N, 7°84'W), Douna (13°21'N, 5°90'W) and Fanzana (13°20'N, 06°13'W). Additional collections were made from Guinea Conakry in 1990: Sombili and Timbi Madina (11°24'N, 12°16'W); from Senegal in two different years (2002: Kedougou, 12°14'N, 12°36'W; and 2006: Jingoreh Maffy, 13°41'N, 13°39'W; Wassadou, 13°21'N, 13°21'W; Samecouta, 12°36'N, 12°08'W); and Cameroon in 2005: Doujouf (10°34'N, 14°17'E), Sanguere Ngal (9°12'N, 13°30'E), Bodova (9°59'N, 14°10'E), and Tongo (8°55'N;13°31'E). Specimens were sorted morphologically to *A. gambiae s.l*. and by gonotrophic stage. Ovaries of half-gravid females were dissected and placed in numbered individual micro-tubes containing modified Carnoy's solution (1:3 glacial acetic acid: 100% ethanol). Carcasses were placed in correspondingly numbered micro-tubes over desiccant.

### Cytological determination of karyotype

Polytene chromosomes were prepared following [13] with slight modifications. A portion of one ovary (1/4–1/3) was placed into a drop of 50% propionic acid deposited on a slide, where it was allowed to swell to approximately twice the original size (2–5 min). After adding another drop of propionic acid, follicles were freed with a dissecting needle. A fresh drop of propionic acid was added and a coverslip was placed on the tissues. The assembly was sandwiched between filter paper, coverslip side up. The back of a pen or a pencil (with eraser) was used to gently tap on the coverslip to break the follicles and spread the chromosomes, taking care to avoid movement of the coverslip. Chromosomes were flattened by blotting the assembly between two layers of filter paper and pressing firmly but carefully with the palm of the hand. The slide was checked using a phase contrast microscope (Olympus BX40) using 20×, 40× or 100× objectives. The 2Rj arrangement was interpreted based on the cytogenetic map of *A. gambiae *polytene chromosomes created by M. Coluzzi and V. Petrarca (see *A. gambiae *poster from Science 298, 4 October 2002; [14]). Excess ovarian tissue was retained for re-examination in case of discrepancy. For the same purpose, slide preparations were sealed with lactic acid and stored at -20°C.

### PCR-based determination of karyotype

Genomic DNA was isolated from individual mosquitoes using one of the following: DNeasy Extraction Kit (Qiagen, Valencia, CA), Puregene kit (Gentra Systems, Inc., Minneapolis, MN), DNAzol kit (Molecular Research Center, Inc., Cincinnati, OH.) or Easy-DNA kit (Invitrogen, Carlsbad, CA). *Anopheles gambiae s.s*. and its molecular forms were identified using one of two rDNA-based PCR/RFLP assays [15] or [16]. That of Santolamazza *et al *[16] was preferred due to the longer shelf-life of restriction enzyme *Mse*I versus *Hha*I (New England Biolabs, Ipswich, MA) observed in this study.

**Figure 1:**
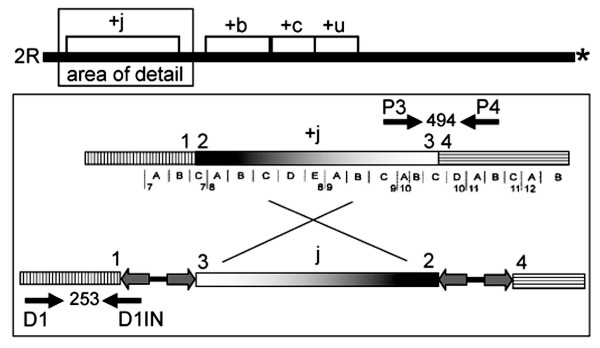
Strategy employed for PCR-based karyotyping of the 2Rj inversion. At top, schematic representation of 2R (black line), indicating approximate position of inversions j, b, c and u; asterisk indicates centromere. Below, schematic representation of primer binding sites on the standard (+^j^) and inverted (j) chromosomes (hatched and shaded rectangles). The relevant portion of the cytogenetic map is presented below the standard chromosome. Numbers above each chromosome refer to different breakpoint junctions. Double-headed arrow between breakpoint junctions of the inverted chromosome represents an identical 14.6 kb insertion containing 5.3 kb inverted repeats separated by a 4 kb spacer. Labeled arrows above and below chromosomes indicate primer pairs targeting the standard and inverted arrangement of 2Rj, respectively; expected length of the PCR product in bp given between the arrows. Chromosome segments and primers are not to-scale.

**Figure 2:**
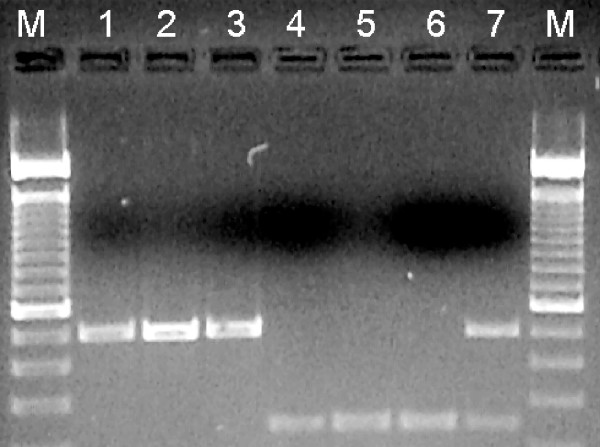
Application of molecular karyotyping to *A. gambiae *from Mali. Electrophoretic separation of PCR products from 2R+^j^/+^j ^(lanes 1–3), 2Rj/j (lanes 4–6) and 2R+^j^/j (lane 7) specimens. Upper and lower bands are 494 bp and 253 bp, respectively. M, 100 bp ladder (Life Technologies, Rockville, MD).

Prospective primer pairs were designed to amplify across the breakpoints of the standard (2R+^j^) or inverted (2Rj) arrangement by importing sequences corresponding to the breakpoint junctions from the *A. gambiae *reference genome (PEST, 2R+^j^) or from *A. gambiae *BKO [12] into Primer3 [17]. The four primers employed in the final 2Rj karyotyping assay included one pair targeting the 2R+^j ^proximal breakpoint (P3, 5'-CGAAGAGGAAGTCGTGCTTT and P4, 5'-GTTGACGTTTTGGGTCGTTT) and another pair targeting the 2Rj distal breakpoint (D1, 5'-GCGTTGTCAATAATGCCTGA and D1IN, 5'-TATTGGGTTTTTACCCCATAATC) (see Figure 1). PCR was carried out in a 25 μl reaction containing 5–7.5 pmol of each primer, 20 mM Tris-HCl (pH 8.4), 50 mM KCl, 1.5–2.0 mM MgCl_2_, 200 μM each dNTP, 1 U of Taq polymerase, 4.8% DMSO, 1% BSA and 1 μl of the template DNA. The PCR conditions were 94°C for 2 min, 40 cycles of 94°C for 20 s, 55°C for 15 s and 72°C for 20 s followed by 72°C for 5 min and a 4°C hold. PCR products were separated on 1.5% agarose gels stained with ethidium bromide (see Figure 2).

For the samples collected from Senegal in 2006, DNA was extracted independently at the MRC and the University of Rome, and PCRs were run in parallel as a test of reproducibility and robustness of the assay. The samples collected from Mali were analysed exclusively at the University of Notre Dame, and the remaining samples were analysed exclusively at the University of Rome.

## Results

### Assay design

The 2Rj inversion breakpoints were completely characterized [12] based on sequence determined from a BAMAKO colony (BKO) derived from Moribabougou, Mali and comparison with the 2R+^j ^*A. gambiae *PEST reference genome. Both breakpoint junctions of inversion 2Rj contain identical 14.6 kb insertions, each of which consists of long inverted repeat arms separated by a spacer. This large structure precluded any molecular karyotyping strategy for the inverted chromosome that would require PCR amplification across the complete breakpoint junction (*i.e*., from regions 1–3 or 2–4 in Figure 1). Moreover, although these structures have not been found elsewhere in the genome, they are composed of degraded remnants of transposons and other repetitive elements, making them poor targets for specific amplification. Accordingly, a strategy was adopted in which the 2Rj-specific primer (D1IN) was designed to anneal at its 5'-end to the last 15 bp of the inverted repeat while anchored by 8 bp at its 3'-end to the single-copy chromosomal DNA of region 1 that flanks the distal breakpoint (see Figure 1). To reduce the problem of primer interference common to multiplex PCR when a universal primer is shared by two PCR products, the 2R+^j ^standard arrangement was detected by an independent pair of primers (P3 and P4). These primers amplify a 494 bp product spanning regions 3–4 that is unique to the uninverted arrangement, as region 3 is no longer adjacent to region 4 on the inverted chromosome (Figure 1). The 494 bp product from the 2R+^j ^standard arrangement is easily distinguished by gel electrophoresis from the 253 bp product specific for the 2Rj inversion amplified by D1IN and its partner D1 (Figure 2).

### Validation in BAMAKO and SAVANNA populations from Mali

Collections of half-gravid *A. gambiae *were conducted in southern Mali during August-September 2004, targeting seven villages near the Niger River and its tributaries where the BAMAKO form was predicted to occur. Of the 968 successfully karyotyped specimens, only six 2Rj heterozygotes (0.6%) were found among 553 2R+^j ^standard and 409 2Rj inverted homozygotes. Of the 2Rj inverted homozygotes, 377 (92%) carried BAMAKO karyotypes: jcu with b polymorphic.

The 2Rj multiplex assay was conducted on DNA extracted from 550 of the 968 karyotyped specimens, chosen from the seven villages such that the representation of 2Rj standard and inverted arrangements was approximately equal. Correspondence between cytological and molecular determination of the 2Rj karyotype was high (≥ 99%) for both homokaryotypes (Table 1). In the case of 2Rj standard homozygotes, only one discrepancy was found among 306 successful amplifications: the molecular pattern indicated a 2Rj heterozygote instead of a standard homozygote. Whenever discrepancies occurred between cytological and molecular determinations, standard practice was to repeat the assay under three conditions that included (1) all four primers together, (2) only 2R+^j ^primers P3 and P4, and (3) only 2Rj primers D1 and D1IN. This procedure gave insight into whether the initial result was repeatable, and which arrangement might be responsible for the anomalous results. Upon repetition, the molecular results were unchanged; the correct size bands also appeared in reactions containing only 2Rj or 2R+^j ^primers. Although the chromosome preparation was not available for validation, a true inversion heterozygote would be difficult to miss cytologically. Nevertheless, human error could have resulted in mistaken data entry, dissociation of carcass and chromosomes into tubes with different numbers, or DNA contamination leading to disagreement between the two methods. The rate of such error is acceptably low, as correspondence between molecular and cytological determinations of 2Rj standard homozygotes was nearly 100% (305/306).

**Table 1:** Proportion of molecular karyotypes that matched the cytologically determined arrangement of inversion 2Rj in natural populations of *Anopheles gambiae s.s*.

	Country Sampled				
		
Karyotype	Senegal (n = 147)	Guinea Conakry (n = 18)	Mali (n = 550)	Cameroon (n = 17)	Combined total (n = 732)
2Rj/j total	23/23 (100%)	2/2 (100%)	238/241 (98.8%)	--	263/266 (98.9%)
2Rj/j SAV	23/23 (100%)	1/1 (100%)	16/16 (100%)	--	40/40 (100%)
2Rj/j BAM	--	1/1 (100%)	222/225 (98.7%)	--	223/226 (98.7%)
2R+^j^/+^j^	56/57 (98.2%)	6/6 (100%)	305/306 (99.7%)	13/13 (100%)	380/382 (99.5%)
2Rj/+^j^	40/67 (59.7%)	7/10 (70%)	3/3 (100%)	2/4 (50%)	52/84 (61.9%)

SAV, SAVANNA chromosomal form; BAM, BAMAKO chromosomal form.

Among 241 2Rj inverted homozygotes that were successfully amplified by PCR, 238 (98.8%) yielded the 253 bp band in agreement with traditional karyoype analysis. Two of three exceptions yielded a single band larger than 253 bp, but slightly smaller than the 494 bp band expected of standard chromosomes. In both cases, it was possible to verify the chromosomal banding pattern preserved on slides, which was confirmed as 2Rj inverted. Thus, the apparent discrepancy between molecular and cytogenetic determinations could be due to small insertions on the inverted chromosome. The third exception produced a single band indistinguishable in size from that expected of standard chromosomes. Unfortunately the chromosome preparation was unavailable for validation, so an error in recording or interpretation of the banding pattern cannot be ruled out.

Of only 6 2Rj heterozygotes sampled in Mali, two did not amplify and one was lost. The remaining three were successfully scored as 2Rj/+^j ^heterozygotes by the multiplex PCR assay.

### Validation in SAVANNA populations beyond Mali

The geographic distribution of the 2Rj inversion greatly exceeds that of the BAMAKO chromosomal form, where it is associated with the cu and bcu arrangements in populations along Niger River in southern Mali and northern Guinea Conakry [18]. Beyond this limited region, the 2Rj inversion is associated with other arrangements such as b, d and bd as far west as Guinea Bissau [9], Senegal [19] and eastward into Cameroon, Central Africa [20]. To test the molecular diagnostic across this broader range, particularly from samples in which 2Rj is expected to show higher levels of polymorphism, collections were made from Senegal, western Guinea Conakry and Cameroon.

A total of 181 karyotyped SAVANNA specimens and one karyotyped BAMAKO specimen from the three countries were successfully amplified for molecular karyotype analysis of 2Rj (Table 1). Of these, 76 were 2R+^j ^standard homozygotes and all except one (98.7%) yielded the expected ~500 bp standard band. The exception, a cytologically 2R+^j ^standard specimen collected in Kedougou, Senegal in 2006, was classified as a heterozygote molecularly. This result was confirmed not only by repetition of the molecular diagnostic with primer pairs applied separately and in combination (as described above), but also by different laboratories (at the University of Rome and the MRC) on DNA extracted independently from different portions of the mosquito. It is therefore unlikely that the discrepancy resulted from contamination of primers and template, but other sources of human error cannot be ruled out. An additional 25 specimens from Senegal and Guinea Conakry were 2Rj inverted homozygotes, all of which (100%) revealed the expected ~250 bp inverted band.

In contrast to the highly congruent molecular and cytological results for 2Rj standard and inverted homozygotes, only ~60% (49/81) of heterozygotes from Senegal, Guinea Conakry and Cameroon were scored as heterozygotes molecularly. The results of three specimens were conservatively included among the molecular "miscalls" despite the fact that two molecularly distinct bands were seen following gel electrophoresis, because one of the two bands was an unexpected size. When the PCR was conducted using primer pairs for standard and inverted arrangements separately, two of these "miscalls" were evidently due to insertions ≥ 400 bp between the standard primers (P3+P4) while the remaining one seemed to be caused by an insertion of ~400 bp between the inverted primers (D1+D1IN). More definitive resolution of these three exceptions requires sequence determination, a step not taken in this study. An additional two of the molecular "miscalls" from Senegal samples collected in 2006 were placed in this category because parallel analyses run at MRC and the University of Rome were in disagreement and could not be resolved despite independent DNA extraction and repeated trials. Whereas one institution correctly scored a specimen as a 2Rj/+^j ^heterozygote, the other scored the same specimen as 2R+^j^/+^j ^standard (all other results from the 2006 Senegal samples were identical between the two institutions). The vast majority of discordant cytological and molecular results (27 of 32 miscalls, 84%) were due to a cytological heterozygote being scored molecularly as a 2R+^j ^standard. Repetition of the assay with separated primer pairs demonstrated that this outcome reflected the failure of the D1+D1IN primer pair to amplify the 2Rj inverted arrangement.

## Discussion

A PCR diagnostic assay that detects and differentiates both 2Rj arrangements has been developed and extensively validated. In all samples, results from the molecular assay agree with traditional cytological karyotype determination in 99% (643/648) of tests in which the specimen analysed was a 2Rj standard or inverted homozygote. By contrast, only 62% of cytologically scored 2Rj/+^j ^specimens (52/84) were correctly scored as heterozygous. This result is consistent when considering the Senegal, Guinea Conakry and Cameroon data (Table 1); the size of the Malian sample is too small to be reliable. A possible explanation for the low specificity of the PCR test for heterozygotes could be the presence of null alleles (mutations that preclude primer binding) on the 2Rj inverted chromosome, as the majority of miscalls resulted from failure of the inverted chromosome to amplify. This proposal is not unrealistic in light of the estimated rate of null alleles in *A. gambiae *(~5%; [21,22]), and considering both the geographic distance between sampling locales and the fact that the primers were designed based on sequences determined from Malian specimens. In theory, mosquitoes that carry 2Rj null alleles on both inverted chromosomes (which would result in amplification failure) should be rare relative to mosquitoes that carry only one 2Rj null allele (which would result in the anticipated outcome from 2Rj inverted homozygotes and exclusive amplification of the standard chromosome in 2Rj heterozygotes). This explanation can easily account not only for the relatively large incongruence between cytological and molecular methods as applied to 2Rj heterozygotes, but also for fact that nearly all misclassifications were molecularly typed as 2Rj standard homozygotes. Whether or not this explanation applies equally to 2Rj heterozygotes in the Niger River zone in southern Mali relates to the possibility of substructure between BAMAKO and other *A. gambiae *populations within and outside of this zone, as well as the question of differential selection pressures on this inversion in BAMAKO versus other taxa. These are questions that should be addressed by future studies, but the issue is not a matter of practical importance in the implementation of this assay, as heterozygote prevalence is so low in this region.

The development of a molecular karyotyping assay for 2Rj was motivated by the lack of practical tools for the rapid identification of the BAMAKO chromosomal form. The validation efforts described here demonstrate that this assay will serve that purpose very reliably, at least in southern Mali near the Niger River.

In the total sample of nearly 1000 karyotyped specimens from Mali, only 6 2Rj heterozygotes were found. This pattern is not due to a Wahlund effect created by pooling samples across different villages, as it is applies equally well to a sub-sample from the village of Kela. Of 608 karyotyped mosquitoes, only 4 2Rj heterokaryotypes were found and a highly significant departure from Hardy-Weinberg equilibrium in the direction of heterozygote deficit was detected (Table 2). Given that BAMAKO is characterized by j homozygotes (jcu/jcu, jbcu/jcu, jbcu/jbcu) and that j segregates at a very low frequency in SAVANNA populations from Mali, these data confirm the hypothesis of assortatively mating SAVANNA and BAMAKO chromosomal forms within the S molecular form in southern Mali. Indeed, instances in which the j inversion is associated with non-BAMAKO karyotypes in Kela are rare (30/364; 8%) and it is possible that some or all could represent "hybrids" between BAMAKO and SAVANNA forms [4].

**Table 2:** Test for Hardy-Weinberg equilibrium on 2Rj karyotypes sampled from Kela, Mali during August-September 2004.

Karyotype	N	%	X_1_^2^	P
2R+^j^/+^j^	o 243	40.03		
	e 98.89	16.29		
2R+^j^/j	o 4	0.66	590.50	0.0000
	e 292.22	48.14		
2Rj/j	o 360	59.31		
	e 215.89	35.57		

## Conclusion

As anticipated by Coluzzi [1], the *A. gambiae *reference genome has greatly facilitated study of the ongoing evolution and diversification of this medically important taxon. A case in point is the molecular characterization of the 2Rj inversion breakpoints and the ensuing development of a PCR-based assay for its rapid karyotyping. The intensive efforts at field validation reported here discourage the application of this assay for molecular karyotyping of j-carriers of the SAVANNA chromosomal form. On the other hand, molecular karyotyping of 2Rj is a robust and reliable method to identify BAMAKO in West African sites where this chromosomal form is prevalent and sympatric with a SAVANNA form having a low frequency of 2Rj-carriers. Moreover, cytogenetic analysis of karyotypes from southern Mali is consistent with previous proposals that BAMAKO mates assortatively, raising the possibility that BAMAKO may be in the early stages of speciation within the S form of *A. gambiae*. Until now only the ovarian polytene chromosome banding pattern was available for recognition of the BAMAKO form, severely limiting its study – especially at the larval stages. Thus, the special significance of the 2Rj karyotyping assay reported here is that it offers for the first time a method to rapidly detect BAMAKO at all stages and both sexes. Application of this assay offers the opportunity to deepen understanding of the genetic, ecological and behavioral processes driving ongoing adaptation and diversification of *A. gambiae*.

## Authors' contributions

MBC participated in the design of the study, led the field collections in Mali, participated in molecular and cytological karyotyping, data analysis and drafting the manuscript. MP participated in molecular karyotyping of specimens from Senegal and Guinea Conakry, and participated in molecular and cytological karyotyping of the Cameroon sample. BC participated in field collections of specimens from Senegal, and in their molecular and cytological karyotyping. DN participated in molecular karyotyping of specimens from Senegal. MJ, LK, and ID coordinated and led field collections in Senegal and Guinea Conakry. AF participated in cytological karyotyping of specimens from Mali. MK participated in molecular karyotyping of specimens from Mali. VP participated in the cytological karyotyping of samples from Senegal and Guinea Conakry. FS, DC, AdT, and ST helped design and coordinate the study. In addition, AdT and VP helped interpret data and draft the manuscript. NJB participated in design and coordination of the study, data analysis, and drafting the manuscript. All authors read and approved the final manuscript.
